# Deer do not affect short‐term rates of vegetation recovery in overwash fans on Fire Island after Hurricane Sandy

**DOI:** 10.1002/ece3.5674

**Published:** 2019-10-06

**Authors:** Chellby R. Kilheffer, H. Brian Underwood, Jordan Raphael, Lindsay Ries, Shannon Farrell, Donald J. Leopold

**Affiliations:** ^1^ College of Environmental Science and Forestry State University of New York Syracuse New York; ^2^ US Geological Survey Patuxent Wildlife Research Center College of Environmental Science and Forestry State University of New York Syracuse New York; ^3^ National Park Service Fire Island National Seashore Patchogue New York

**Keywords:** *Ammophila breviligulata*, barrier island, imagery classification, random forest classification, resilience, white‐tailed deer

## Abstract

1. Coastal resilience is threatened as storm‐induced disturbances become more frequent and intense with anticipated changes in regional climate. After severe storms, rapid recovery of vegetation, especially that of dune‐stabilizing plants, is a fundamental property of coastal resilience. Herbivores may affect resilience by foraging and trampling in disturbed areas. Consequently, assessing the impacts of herbivores on recovering vegetation is important for coastal land management.
2. We combined imagery classification, wildlife monitoring, and trend analysis to investigate effects of white‐tailed deer on recovery rates of vegetation four years poststorm in nine overwashed areas. We estimated local deer density with trail cameras, how it relates to an index of primary productivity, and assessed the relationship between deer density and rates of vegetation recovery in overwash fans.
3. Prestorm vegetation cover consisted of shrubs and sporadic patches of beach grass. Poststorm cover was dominated by beach grass. At current rates, vegetation coverage will return to prestorm conditions within the decade, though community transition from grasses to shrubs will take much longer and will vary by site with dune formation.
4. The effect of deer on rates of vegetation recovery was negative, but not statistically significant nor biologically compelling. Although effects of deer trampling on beach grass are evident in classified imagery, deer foraging on beach grass had little effect on its rate of spread throughout overwash fans.
5. While the rate of spread of the primary dune‐building grass was not deleteriously affected by deer, locally high deer densities will likely affect the future establishment and development of herbs and shrubs, which are generally more palatable to deer than beach grass.

## Graphical abstract

A dense white‐tailed deer population did not affect the rate of dune vegetation recovery after a large‐extent hurricane. Deer may, however, affect establishment and development of palatable plant species as disturbed areas continue to recover.

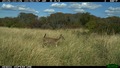

## 1 INTRODUCTION

Barrier islands have persisted for thousands of years despite frequent disturbance (Ehrenfeld, 1990; Feagin et al., 2010; Snyder & Boss, 2002). During storm events, foredunes along the ocean‐coast of barrier islands erode and serve as natural defense against disruption of inland ecosystems (Durán & Moore, 2013; Hapke, Brenner, Hehre, & Reynolds, 2013; Sallenger, 2000). Where erosion rates are high, vegetation communities often remain disturbed and vulnerable to future storms (Roman & Nordstrom, 1988). In the absence of subsequent disturbance, a positive feedback between plants and sand entrapment begins to form a new foredune (Maun, 2009; Vinent & Moore, 2015).

Postdisturbance vegetation growth mediates dune formation and subsequent coastal vulnerability to future storm impacts (Brantley, Bissett, Young, Wolner, & Moore, 2014; Durán & Moore, 2013; Olson, 1958; Pendleton, Williams, & Thieler, 2004; Vinent & Moore, 2015). In the northeastern United States, *Ammophila breviligulata*, American beach grass, is the dominant plant species responsible for foredune growth (Maun, 1985, 2009; Stuckey & Gould, 2000) since it is tolerant of salt spray and its growth is enhanced by sand burial. Growth and expansion rates of *A. breviligulata* are often quite high in areas with consistent sand deposition (Kent, Owen, Dale, Newnham, & Giles, 2001; Maun, 2009; Olson, 1958). Consequently, annual mapping of the expansion of *A. breviligulata* and other plants after a disturbance can increase our understanding of the factors that impinge on vegetation recovery and aid in developing potential mitigation strategies. Mapping plant cover is facilitated through use of digital imagery and digital classification methods (Boyle et al., 2014; Kilheffer, 2018).

Factors that affect coastal vegetation recovery rates, including herbivory (Carruthers et al., 2013; Keiper, 1990; Seliskar, 2003) and trampling (Bowles & Maun, 1982; Santoro et al., 2012; Šilc, Caković, Küzmič, & Stešević, 2017), have the potential to also affect resilience to future storms (Houser et al., 2015; Vinent & Moore, 2015). In this manuscript, we define resilience as the system's ability to respond to disturbance without losing ecological function (Klein, Smit, Goosen, & Hulsbergen, 1998). Due to the dynamic nature of dune systems, multiple states of equilibrium often exist after a disturbance (Zinnert, Stallins, Brantley, & Young, 2016), so resilience captures the ability of the system to return to a state of equilibrium, though that state may differ from predisturbance conditions.

Hyper‐abundant populations of large herbivores may reduce barrier island resilience by impeding rates of vegetation recovery through the combined effects of browsing, grazing, and trampling. Over the last several decades, populations of native and exotic large herbivores have irrupted to numbers that pose serious challenges to the management of natural resources on barrier islands (Art, 1987; Carruthers et al., 2013; Forrester, Leopold, & Underwood, 2006; Porter, DePerno, Krings, Krachey, & Braham, 2014; Sherrill, Snider, & DePerno, 2010; Wood, Mengak, & Murphy, 1987). Ungulates seek highly productive patches of forage (Bakker, Ritchie, Olff, Milchunas, & Knops, 2006; Bråthen et al., 2007; Lezama et al., 2014; Oksanen, Fretwell, Arruda, & Niemela, 1981; Ritchie, Tilman, & Knops, 1998), especially in nitrogen‐limited environments like most habitats on barrier islands (Ehrenfeld, 1990). In response to variation in net primary productivity, the spatial distribution of herbivores could exert disproportional effects on recovering vegetation communities and compromise barrier island resilience.

The purpose of this study was to better understand the effects of locally abundant white‐tailed deer (*Odocoileus virginianus*) populations on poststorm vegetation recovery rates and, therefore, barrier island resilience. Our main objectives were to (a) estimate vegetation recovery rates for nine overwash fans through use of imagery classification and (b) explore the relationships between local deer density, net primary productivity, and prestorm vegetation cover on poststorm vegetation cover.

## 2 MATERIALS AND METHODS

### 2.1 Study area

**Figure 1:**
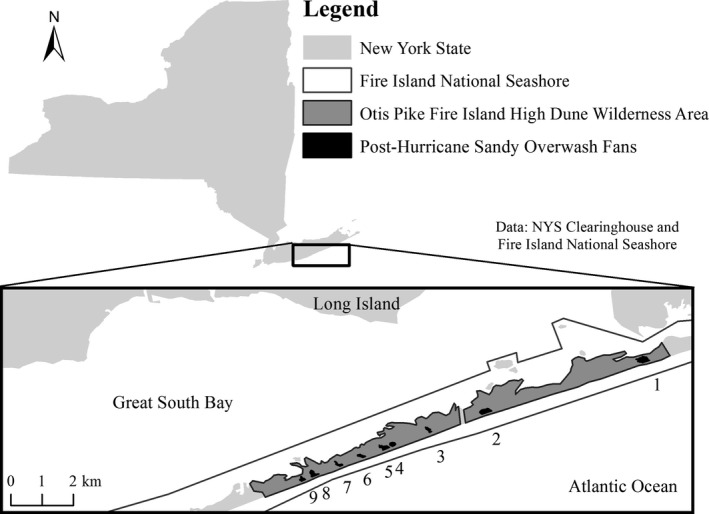
Fire Island National Seashore is located off the southern coast of Long Island, New York, USA, and contains the Otis Pike Fire Island High Dune Wilderness Area

The focal study area is on Fire Island, New York, USA (40.703586 N, 72.952014 W). Fire Island is a barrier island located approximately 6 km from the south shore of Long Island (Figure 1). Fire Island National Seashore is unique within the National Park Service network because it encompasses the Otis Pike Fire Island High Dune Wilderness Area (OPWA), the only federally designated wilderness area in the state of New York. This research focused on the OPWA, which was designated a federal wilderness area in 1980 (FIIS, 2015). On October 29, 2012, Hurricane Sandy, a posttropical cyclone with a massive wind radius (>185 km), caused significant storm surge and inundation of coastal areas along the northeastern United States (Blake, Kimberlain, Berg, Cangialosi, & Beven, 2013; Hapke et al., 2013), including a breach of Fire Island. Before Hurricane Sandy, foredunes in the OPWA were 4–15 m high (Hapke et al., 2010). Many stretches of foredune in Fire Island's OPWA were overwashed by Hurricane Sandy's high storm surge, which deposited large volumes of sand inland, effectively removed the foredune, and left overwash fans ranging in size from 0.60 to 3.24 ha.

Deer in the OPWA have consistently exhibited densities of approximately 25 ± 8 deer/km^2^ since the mid‐1980s based on aerial and ground surveys (O'Connell & Sayre, 1989; Underwood, 2005). Trampling by deer results in structural damage to above‐ground and below‐ground vegetation, leaving a legacy network of trails lacking vegetation throughout the OPWA (NOAA, 2012; NYSC, 2010). Deer graze grasses and herbs in overwash fans of the OPWA (Kilheffer, 2018). However, the degree to which deer exert an impact on the composition, structure, and recovery rate of recovering vegetation in overwash fans after storms like Hurricane Sandy remains speculative.

### 2.2 Vegetation cover assessment

We obtained digital orthoimagery for the OPWA before Hurricane Sandy (NYSC, 2010), in the aftermath of Hurricane Sandy (NOAA, 2012), and in the third and fourth growing seasons after Hurricane Sandy (AG, 2015; NYSC, 2016). Imagery resolutions were between 0.15 and 1 m, and we used ArcGIS tools to mosaic orthoimagery tiles into one contiguous image (version 10.5; ESRI, Redlands, CA). We used random forest (RF) image classification to characterize vegetation cover in and around nine post‐Hurricane Sandy overwash fans in the OPWA. The RF method is widely considered the most accurate machine‐learning technique available (Breiman, 2001; Cutler et al., 2007; Xie, Sha, & Yu, 2008). Random forest uses multiple classification trees in a regression framework to classify land cover from spectral, geographic, or other user‐defined raster layers. Similar to maximum likelihood classification methods, the user trains samples from imagery (Lillesand, Kiefer, & Chipman, 2014). Instead of strictly using spectral information to identify land cover, classification trees use linear combinations of input layers and decision trees to partition the image into regions that are increasingly homogeneous.

We identified 20 visually distinct land cover categories over the entire mosaicked image for each year to account for differences in atmospheric correction and spectral signature. We used the *Train Random Trees* classifier in ArcGIS to define classification trees using red, green, blue, and near‐infrared spectral bands (Kilheffer, 2018) and smoothed boundaries between classes with the *Boundary Clean* function in ArcGIS. We then aggregated land cover into water, sand, marsh grass, beach grass, shrubs, and trees.

We assessed classification accuracy by calculating confusion matrices of user's and producer's accuracies proportionally for each class (*n* = 500 points) in each year and kappa statistics (Stehman & Czaplewski, 1998; Van Deusen, 1996; Xie et al., 2008). User's accuracy measures the probability that a randomly selected point classified in a category actually depicts that category on the ground. Producer's accuracy measures error of omission by comparing the number of points classified in a category to the number of points actually within that category in the image (Congalton, 2005). Kappa statistics between 0.61 and 0.80 indicate substantial agreement between the classified image and true land cover, and >0.81 are believed to be nearly perfect (Landis & Koch, 1977).

Finally, we compared total classified vegetation cover with that estimated from a lattice of 1‐m^2^ plots extending the length and width of each overwash fan (Kilheffer, 2018). We regressed average vegetation cover in each overwash fan on total classified vegetation cover pooled between 2015 and 2016. We tested the statistical hypothesis of a slope not significantly different from unity and intercept not significantly different from zero to assess potential bias in recovery rate estimation because imagery was captured in spring before the growing season when field data were collected.

### 2.3 Vegetation recovery rates

We reclassified each image into a binary raster of vegetation cover (grass, shrubs, trees = 1; sand, water = 0). We then used Zonal Statistics in ArcGIS with overwash fan boundaries as zones to calculate the sum of pixels of vegetation cover. We divided the sum of vegetation pixels by the total number of pixels processed in each overwash fan to obtain percent vegetation coverage. We ln‐transformed average percent coverage values for each year and overwash fan and used ordinary linear regression to estimate the exponential recovery rate.

We used the Normalized Difference Vegetation Index (NDVI) as a proxy for vegetation productivity in overwash fans. We obtained Landsat 7 ETM+ Surface Reflectance imagery for the OPWA for the following dates: June 15, 2012, July 04, 2013, June 21, 2014, June 24, 2015, June 26, 2016, and June 29, 2017 (courtesy of United States Geological Survey). Two overwash fans (i.e., 3 and 8) did not have Landsat imagery available on any dates between June 01 and August 30, 2017, so NDVI was calculated from 2012 to 2016 only. Landsat 7 sensors were replete with gaps in data after a Scan Line Corrector failed in 2003 (Andrefouet, Bindschadler, & Colstoun, 2003). To compensate for gaps in imagery data, we used imagery from July 12, 2016 where necessary. We used the Image Analysis tools in ArcGIS to create a composite of the red, green, blue, and near‐infrared bands and calculated NDVI. We calculated NDVI using the following equation:

$$\text{NDVI}=\frac{\text{NIR}-\text{RED}}{\text{NIR}+\text{RED}}$$

NDVI values range from −1 to +1, where negative values typically correspond to pixels with no green vegetation present and positive values correspond to pixels with highly productive (i.e., photosynthetic) green vegetation present (Pettorelli et al., 2005). We used Zonal Statistics in ArcGIS (version 10.2) to calculate average NDVI within each overwash fan.

### 2.4 Local deer density in overwash fans

We used nine Reconyx brand Hyperfire Covert‐IR HC600 cameras to monitor local deer density (i.e., number of deer using each overwash fan) in the OPWA. We programmed trail cameras to take photographs every hour during peak deer activity periods (Kammermeyer & Marchinton, 1977), from 0400 to 1000 and from 1700 to 2300 each day, and to take three photographs at one‐second intervals when motion was detected, including at night using an infrared sensor. We positioned each trail camera near a deer trail crossing in overwash fans. Cameras were monitored biweekly from August to November in 2015 and 2016.

We used methods described by Sanderson and Harris (2013) to calculate total effort per camera, species activity patterns, and density uncorrected for imperfect detection. We created encounter histories for every identifiable deer in each overwash fan from August 1 to November 24 of each year and calculated an estimate of local abundance (Jacobson, Kroll, Browning, Koerth, & Conway, 1997). Identifiable deer were differentiated through (a) presence of a uniquely colored radio collar or (b) antler points and conformation for males. We used the “species abundance by location by year by month” from DataAnalyze (Sanderson & Harris, 2013) as the total number of deer observations. We calculated a population factor as the proportion of uniquely identifiable deer to the number of identifiable deer observations. We assumed the proportion of times that identifiable deer appeared in overwash trail cameras was the same for unidentifiable deer over the same duration. We calculated the total number of deer in each overwash fan using the population factor and the total number of deer recorded in camera images, including deer that were not uniquely identifiable. We predicted total abundance for two overwash fans during 2015 with no identifiable deer from a regression of total abundance on the numbers of deer recorded in camera images from 2016. We then divided total abundance of deer into the area of each overwash fan to obtain local deer density. We treated local deer density as representing the average number of resident animals using each overwash fan during the growing season and not as global estimates as many deer used more than one overwash fan.

We used the *lm* function in R (R Core Team, 2013) to assess the relationship between local deer density and average NDVI for each overwash fan in 2015 and 2016 through analysis of covariance. Finally, we used the general linear model (i.e., *glm* in R) to assess the relationship between average local deer density between years and pre‐Sandy (2010) vegetation cover on the estimated rates of vegetation recovery for each overwash fan. We assessed model fit using analysis of residuals, inspection for outliers, and coefficient of determination (*R* ^2^). Equations for these two analyses are as follows:

$$\begin{array}{c} lm(\text{DeerDensity}∼\text{Year}+\text{NDVI}+\text{Year}\times \text{NDVI})\\ glm(\text{RecoveryRt}∼\text{InitialCover}+\text{AvgDeerDensity}) \end{array}$$

## 3 RESULTS

### 3.1 Vegetation cover assessment

**Figure 2:**
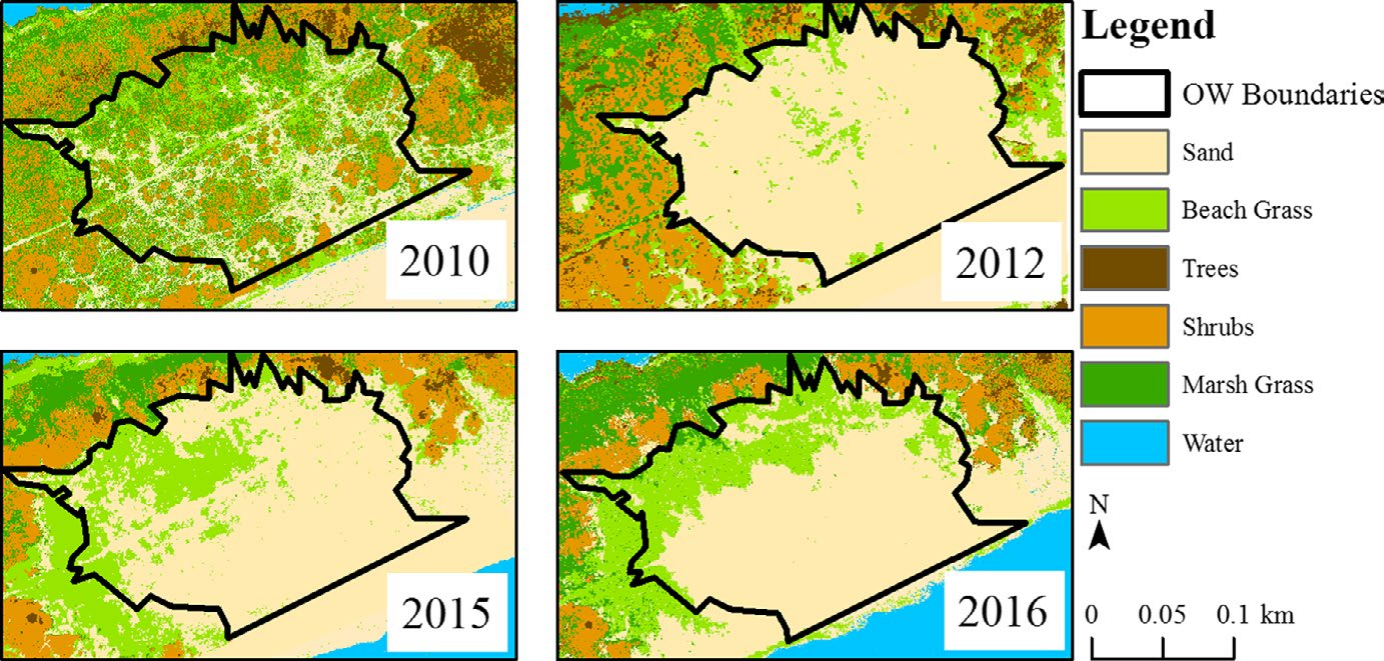
Image classifications for Overwash fan #1, an area overwashed by Hurricane Sandy in the Otis Pike Fire Island High Dune Wilderness Area, New York. Trails where vegetation does not grow, predominantly as a result of deer trampling, are evident in prestorm (2010) and recovering (2015, 2016) imagery in overwash fans

Random forest classification of satellite and aerial imagery produced detailed maps of storm‐induced impacts to vegetation and subsequent recovery in overwash fans (Figure 2). Overall accuracy calculated for each year was between 81% and 98%, indicating strong agreement between the classification and visible land cover (Table 1). In 2010, vegetation cover in overwash fans varied between 23.6% and 72.0% (Table 2) and contained mostly shrubs with small patches of *A. breviligulata* (Appendix S1). The regression of average vegetation cover derived from plot sampling on classified cover was significant (*F* = 32.7; *df* = 1, 16; *p* < .0001), predictive (*R* ^2^ = 0.67) and relatively precise (CV = 19.1%). The regression slope was not different from unity (*β*_1_ = 0.97; *SE* = 0.17) but the intercept was significantly different from zero (*β*_0_ = 16.8%; *SE* = 3.3%).

**Table 1:** Accuracy assessments for classified imagery from 2010, 2012, 2015, and 2016 in the Otis Pike Fire Island High Dune Wilderness Area, New York, including user's accuracies (UA), producer's accuracies (PA), overall accuracies (OA), and kappa statistics. Kappa statistics >0.61 indicate substantial agreement between the classified image and true land cover. See text for details

Year	UA	PA	OA	Kappa
2010	0.78	0.79	0.81	0.75
2012	0.90	0.88	0.92	0.89
2015	0.95	0.96	0.98	0.96
2016	0.93	0.89	0.95	0.92

**Table 2:** Percent total vegetation cover from classified digital imagery of overwash fans (OW) in the Otis Pike Fire Island High Dune Wilderness Area, New York, before Hurricane Sandy (2010), in the aftermath of Hurricane Sandy (2012), and in the third (2015) and fourth (2016) growing seasons after Hurricane Sandy and back‐transformed rates of change and initial total vegetation cover (%) in overwash fans among years since Hurricane Sandy

	OW1	OW2	OW3	OW4	OW5	OW6	OW7	OW8	OW9
2010	72.0	61.9	68.8	41.8	55.2	56.1	57.7	59.3	23.6
2012	1.7	1.0	1.5	2.0	1.2	1.9	4.0	1.8	2.3
2015	20.0	6.2	7.9	5.6	13.5	8.1	21.3	21.4	5.7
2016	23.9	12.7	29.6	14.0	22.2	20.1	27.7	37.2	11.3
Slope	2.0	1.9	2.0	1.6	2.1	1.8	1.7	2.2	1.5
Intercept	1.8	1.0	1.4	1.9	1.3	1.8	4.1	1.9	2.2
Year^a^	2018	2019	2018	2019	2018	2019	2018	2017	2019

a Year in which total vegetation coverage (not species composition) is predicted to return to prestorm conditions.

### 3.2 Vegetation recovery rates

Vegetation cover decreased by 96%–99% in areas overwashed by Hurricane Sandy and only small patches of shrubs remained intact after the storm surge. By the third growing season post‐Hurricane Sandy (i.e., 2015), all overwash fans had established vegetation communities dominated by *A. breviligulata* (6%–21% land cover) with small patches of shrubs (<1% land cover). Vegetation coverage continued to increase in 2016 in most overwash fans (11%–37% land cover). However, two overwash fans (i.e., 1 and 2) with slowly recovering foredunes experienced significant inundation and coastal erosion between 2015 and 2016 resulting in roughly one‐quarter of their land areas returning to predominantly bare sand. Exponential rates of vegetation recovery ranged from 1.5% to 2.2% year^−1^ (Table 2).

**Figure 3:**
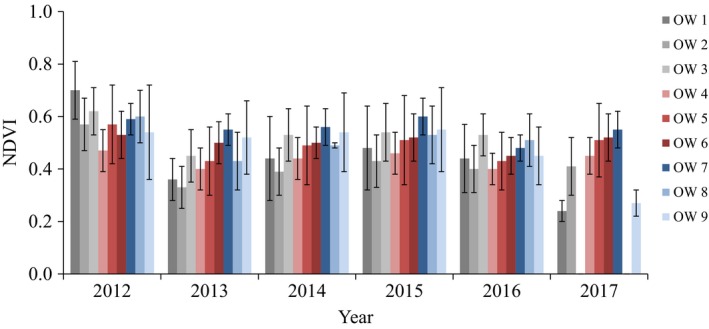
Average Normalized Difference Vegetation Index (NDVI) and standard error bars for nine overwash fans (OW) from 2012 to 2017 in the Otis Pike Fire Island High Dune Wilderness Area, New York. Data are unavailable for overwash fans 3 and 8 in 2017

Net primary productivity, as interpreted from the NDVI, varied among overwash fans. Average NDVI decreased between 2012 and 2013, increased from 2013 to 2015, and decreased from 2015 to 2016 (Figure 3). The maximum and range of NDVI values were greater in 2015 than 2016.

### 3.3 Local deer density in overwash fans

We identified three female and 22 male deer from trail camera photographs taken in 2015, but we were unable to identify individual deer photographed in overwash fans 1 and 2. We calculated between 10 and 39 total deer using each overwash fan in 2015 (Table 3). We identified three adult females and 31 adult males from trail camera photographs taken in 2016, and calculated between 5 and 63 total deer using overwash fans. Averaged across both years, local deer density ranged from 4 to 78 individuals/ha in overwash fans. Average local deer densities appeared higher among overwash fans in 2016 than in 2015 though the effect was not significant (year: *F* = 1.8; *df* = 1, 15; *p* = .21). There was a positive trend in local deer density with increasing NDVI, though the effect was not significant (NDVI: *F* = 3.5; *df* = 1, 15; *p* = .08).

**Table 3:** Local deer densities calculated from trail cameras in overwash fans (OW) in the Otis Pike Fire Island High Dune Wilderness Area, New York, in 2015 and 2016. Many deer were observed in multiple overwash fans, so values are not population estimates

	OW	Number unique deer	Number of obs.	Pop. factor	Total number of deer observed	Number of deer using OW	OW area (ha)	Local deer density (deer/ha)
2015	1	0	0	–	32	19^a^	3.2	6
	2	0	0	–	13	10^a^	2.6	4
	3	5	24	0.21	58	12	0.7	18
	4	3	3	1.00	34	34	1.1	32
	5	6	23	0.26	71	19	1.4	14
	6	8	18	0.44	66	29	0.7	44
	7	11	93	0.12	266	31	0.6	52
	8	10	18	0.56	70	39	1.4	28
	9	9	44	0.20	105	21	0.8	27
2016	1	2	4	0.50	10	5	3.2	2
	2	2	2	1.00	23	23	2.6	9
	3	8	15	0.53	48	26	0.7	38
	4	5	5	1.00	14	14	1.1	13
	5	9	21	0.43	63	27	1.4	20
	6	11	23	0.48	68	33	0.7	49
	7	18	74	0.24	259	63	0.6	104
	8	3	4	0.75	15	11	1.4	8
	9	10	38	0.26	54	14	0.8	18

Note

Number of obs. = Number of unique deer observations.

Pop. factor = Population factor from Jacobson et al. (1997).

Number of deer using OW = Population factor × Total number of deer observed.

Local deer density = Number of deer using OW/OW area.

^a^ Values imputed from regression using the number of unique deer (*Y*) and total number of deer observed (*X*) in each of the other overwash fans.

Linear regression of vegetation recovery rates on pre‐Sandy vegetation cover and local deer density was significant (*F* = 6.5; *df* = 2, 6; *p* = .03, *R* ^2^ = 0.68). Standardized regression coefficients revealed a positive and significant effect of pre‐Sandy vegetation cover (*β* = 0.18; *t* = 3.2; *p* = .02), and a negative but statistically insignificant effect of local deer density (*β* = −0.08; *t* = −1.5; *p* = .19) on vegetation recovery rates.

## 4 DISCUSSION

Prestorm vegetation coverage was the best predictor of poststorm recovery rates in overwash fans created by Hurricane Sandy. We did not find a significant effect of local deer density on the rates of vegetation recovery in storm‐induced overwash fans of the OPWA. While the estimated coefficient associated with local deer density was negative, the effect was neither statistically significant nor compelling. Since Hurricane Sandy, vegetation recovery in overwash fans has increased at an exponential rate of ~2% year^−1^ and coverage is dominated by *A. breviligulata*.

The RF classifier performed adequately in capturing changes in important land cover in and around overwash fans of the OPWA. Plot data recorded on average 16.8% more vegetation cover than image classifications. Imagery used for this study was collected in April before the beginning of the growing season and ground methods were conducted in September during the growing season, so differences in total vegetation cover we observed were unsurprising. Bias in estimated recovery rates was not evident, however, because the regression slope between total cover measured using ground methods and image classification was not different from unity.

Vegetation coverage was greater in 2016 than 2015, but NDVI was greater in all overwash fans in 2015. A drought spanning most of the northeastern United States in 2016 (NOAA, 2016) likely contributed to reduced productivity of *A. breviligulata* in all overwash fans despite increases in cover. Photosynthetic rates of *Ammophila* species are sufficient for growth in water‐limited periods (Alessio, Lillis, Brugnoli, & Lauteri, 2004; Dixon, Hilton, & Bannister, 2004; Gratini, Varone, & Crescente, 2009; Pavlik, 1983), and drought alone did not reduce experimental growth of *Ammophila breviligulata* (Emery, Thompson, & Rudgers, 2010; Marshall, 1965). Marshall (1965) suggested that decline in vigor of *Ammophila arenaria*, a coastal grass closely related to *A. breviligulata*, occurs due to increased competition for water and decreased sand accretion. Disraeli (1984) found significantly lower concentrations of chlorophyll in *A. breviligulata* plants where burial was minimal (e.g., 2.5‐cm) than in areas where burial was great (e.g., 32.5‐cm), and changes in chlorophyll increased exponentially with increases in burial. The presence of budding foredunes on the ocean‐side of overwash fans reduces the amount of sand reaching farther inland (Brenner et al., 2018; Kilheffer, 2018; Maun, 2009), potentially contributing to reduced vigor of *A. breviligulata* in our study. Because NDVI calculates productivity based upon greenness of imagery pixels, a decrease in this productivity index indicates less greenness, or less chlorophyll, in *A. breviligulata* in our sites as foredunes continue to limit sand movement inland (Kilheffer, 2018).

Shrub encroachment in overwash fans has been attributed to the absence of subsequent storm events (Schroder, Hayden, & Dolan, 1979) and is enhanced by dense grass cover where soil nutrients are present (Young, Shao, & Porter, 1995). In a coastal system in Florida, estimated time required for shrub encroachment into *A. breviligulata*‐dominated overwash fans ranged from 19 to 52 years (Johnson, 1997). Because overwash fans 1 and 2 remain highly vulnerable to continued inundation, they may never transition from grass‐dominated to shrub‐dominated communities. In overwash fans that sustain foredune growth, plant communities are likely to return to shrub‐dominated swales with sporadic grasses and herbs over time (Ehrenfeld, 1990; Johnson, 1997; Maun, 2009). However, anthropogenic impacts (i.e., visitors recreating in overwash fans; Lemauviel & Rozé, 2003) and changes in regional climate (i.e., precipitation and temperature patterns; Maun, 2009) could alter the resilience of plant communities and their successional transitions from grasses to shrubs in the OPWA.

The local deer densities we documented in overwash fans were unprecedented, exceeding the average density for the OPWA (Underwood, 2005) by 1–3 orders of magnitude. While we observed more deer in overwash fans exhibiting higher productivity, other factors may have contributed to their utilization by deer. For example, proximity to the salt marsh may facilitate deer utilization of overwash fans (Kilheffer, 2018). Nevertheless, the rate of spread of *A. breviligulata* was not deleteriously affected by deer foraging and trampling in overwash fans. Although we occasionally recorded evidence of deer foraging on green and growing shoots, *A. breviligulata* was mainly consumed by deer during the nongrowing season when more palatable fodder was not readily available (Kilheffer, Underwood, Ries, Raphael, & Leopold, 2019). Gadgil (2002) reported similar findings regarding the effects of herbivores on *A. arenaria*, a closely related beach grass present on European beaches.

Additionally, *A. breviligulata* is very sensitive to the effects of trampling (USDA, 2018) due to sandy substrates. At OPWA, deer trails are clearly evident in pre‐Sandy classified land cover images (Figure 2). Pellerin, Huot, and Côté (2006) found that deer trampling decreased ground vegetation cover, increased coverage of bare peat, and subsequently prevented future establishment of plants in peatlands. Similar legacy effects of vegetation disturbance persist throughout the OPWA from the Burma Road, a long‐abandoned (>50 years) vestige of anticipated development along the island (USNPS, 2016).

Regional climate, a strong driver of barrier island systems (Zinnert et al., 2016), has changed since the Hurricane of 1938, the last catastrophic storm event that caused extensive overwash on Fire Island. Sea level rise has led to increased vulnerability of barrier islands to damage (Horton, Little, Gornitz, Bader, & Oppenheimer, 2015; NAST, 2001; Sallenger, 2000; Vinent & Moore, 2015) by causing storm surges to encroach farther inland than they did only decades ago (Psuty, Grace, & Pace, 2005). Sea level rise in New York has been much greater than the global average (Sallenger, Doran, & Howd, 2012) and evidence suggests that the rate has been increasing (Horton et al., 2015; Psuty et al., 2005). Frequent high water events may hinder vegetation recovery between storms (Vinent & Moore, 2015) and further increase erosion (Houser & Hamilton, 2009), limiting the island's natural resilience.

At current rates, vegetation coverage will return to pre‐Sandy conditions within the decade for most overwash fans. However, transition from grasses to shrubs and small trees will take much longer and depends on how quickly a protective dune forms (Ehrenfeld, 1990; Kilheffer, 2018; Tilman, 1990). Due to the short time frame of our study, we can only speculate on the future development of the vegetation community in overwash fans, especially as regional climate continue to change. Though deer may not impact the rate of *A. breviligulata* recovery in overwash fans, they may ultimately impact the successional trajectory from grasses to shrubs and herbs, particularly if local deer densities remain high. Continued monitoring of plant communities and local deer densities is advised to more fully assess the role of deer in long‐term resilience of Fire Island's wilderness.

## CONFLICT OF INTEREST

The authors do not declare any Conflicts of Interest for this manuscript. Any use of trade, product, or firm names is for descriptive purposes only and does not imply endorsement by the U.S. Government.

## AUTHOR CONTRIBUTIONS

CRK, HBU, SF, and DJL conceived the ideas and defined the methodology; CRK, HBU, JR, and LR collected the data; CRK analyzed the data; CRK and HBU led the writing of the manuscript. All authors contributed fundamentally to the manuscript drafts and gave final approval for its publication.

## Data Availability

Data are archived through the National Park Service Integrated Resource Management Applications (IRMA) portal. IRMA vector dataset code for lattice plot GPS locations: 2264768. IRMA photograph code for lattice plots: 2264762. IRMA Vector Dataset Code for trail camera GPS locations: 2264769. IRMA photograph code for trail cameras: 2264831.
