# Supplementary material for: Deer do not affect short‐term rates of vegetation recovery in overwash fans on Fire Island after Hurricane Sandy
Source: Ecol Evol. 2019 Oct 6;9(20):11742–51. doi: 10.1002/ece3.5674 (PMC6822055; doi:10.1002/ece3.5674)
Supplement: Supplementary file 1 [file ECE3-9-11742-s001.pdf]

Supplementary Materials

Image classifications show changes in vegetation cover from grass and shrubs in 2010 to grass in 2016 as overwash fans in the Otis Pike Fire Island High Dune Wilderness Area, New York recover from Hurricane Sandy. Overwash fan 1 is discussed in the manuscript text.

Overwash fan #2

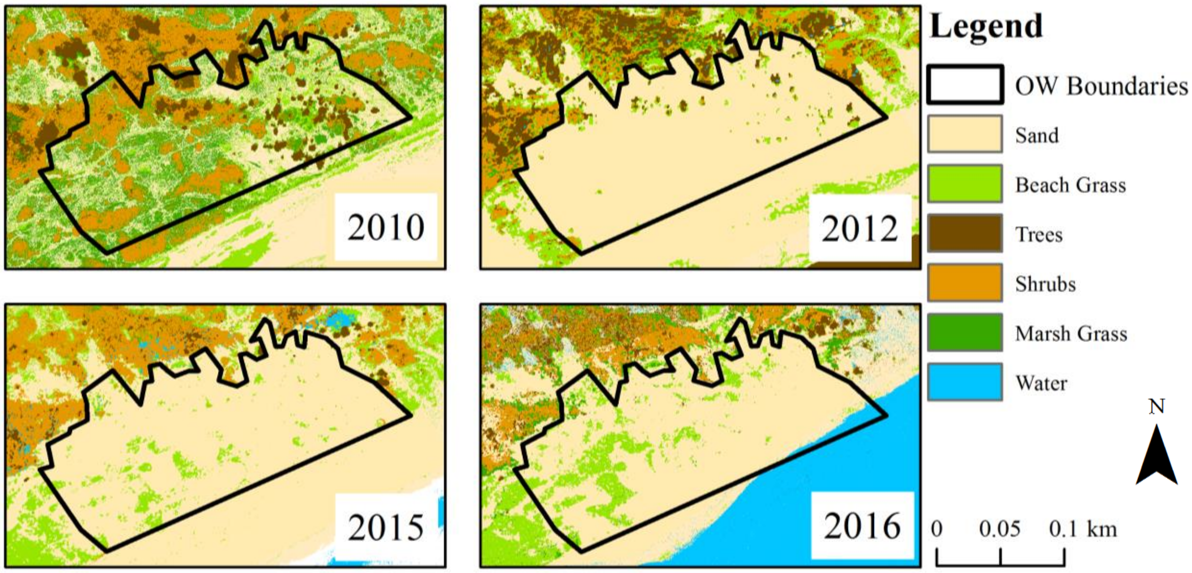

Overwash fan #3

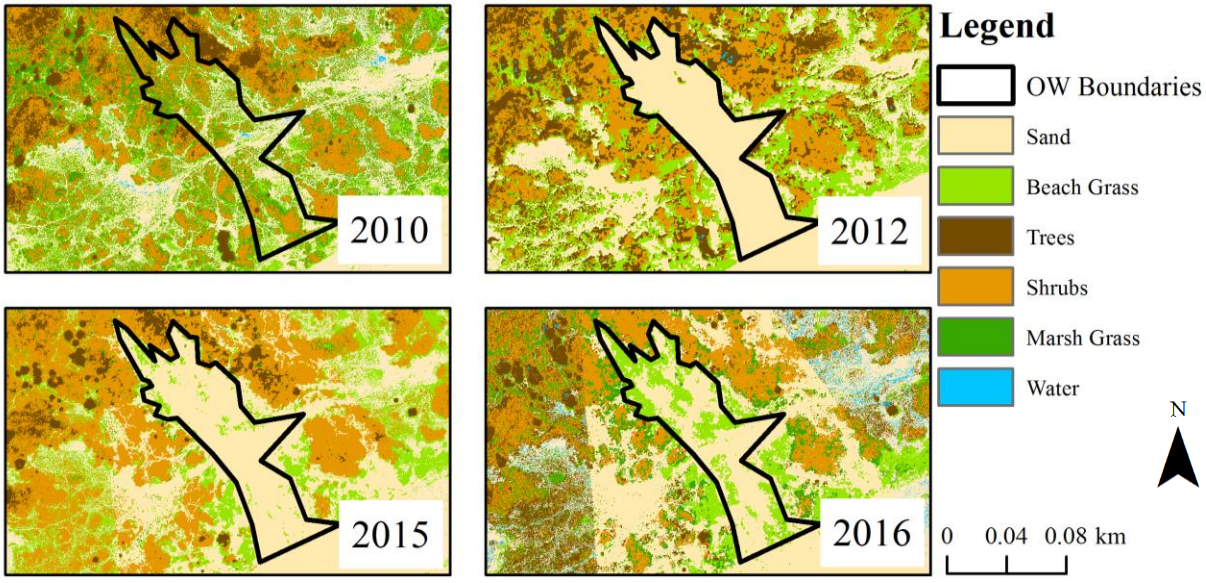

Overwash fan #4

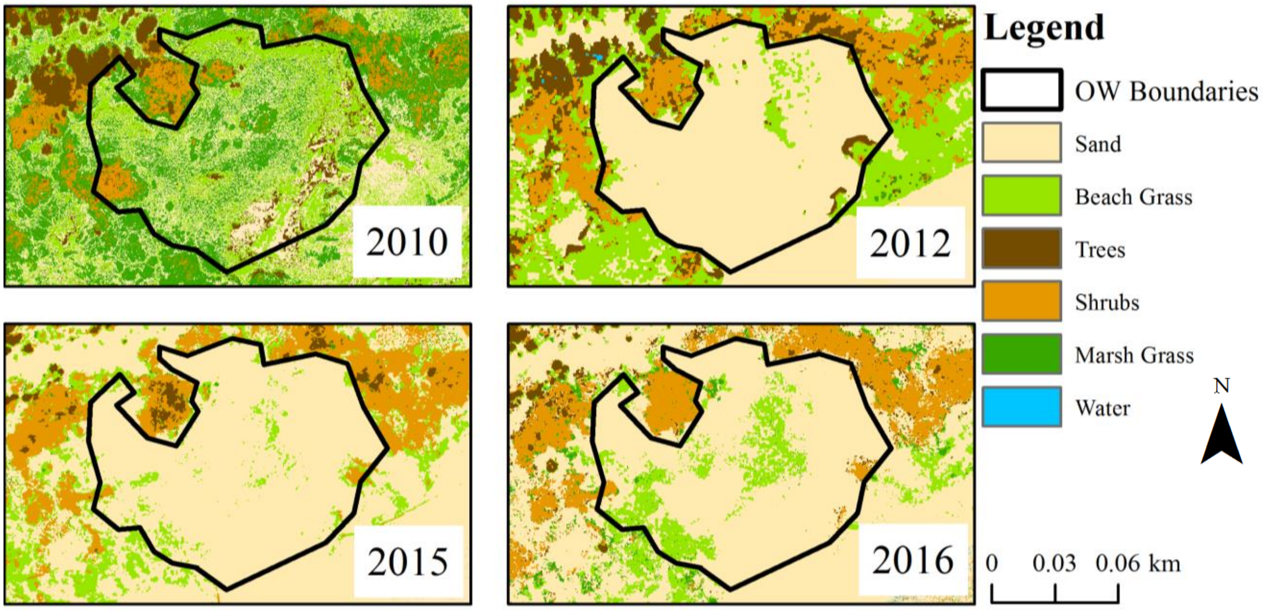

Overwash fan #5

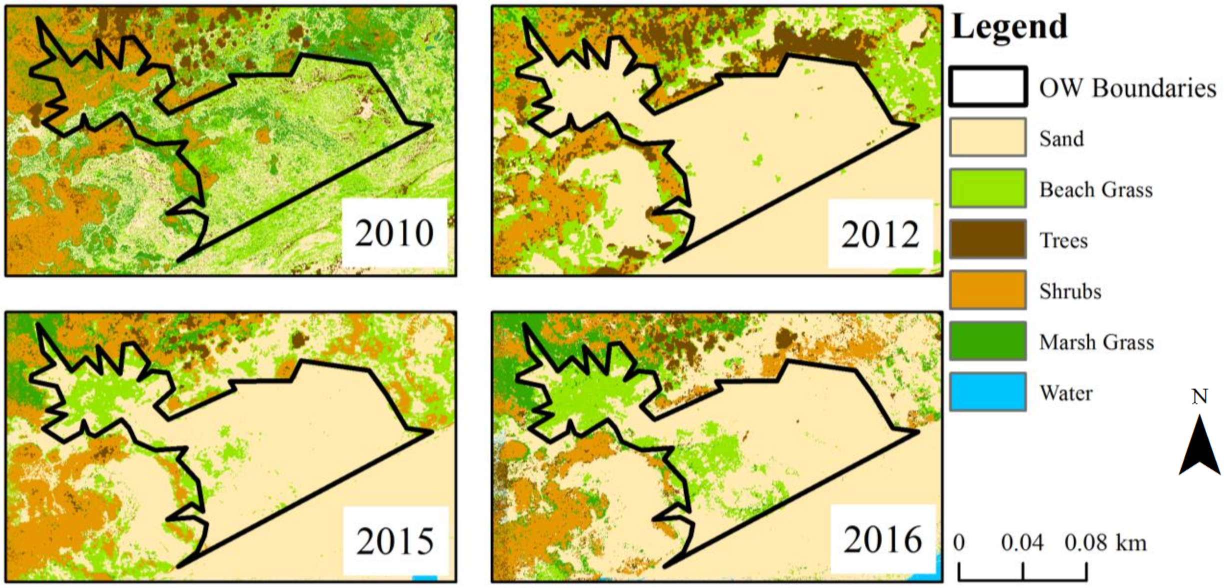

Overwash fan #6

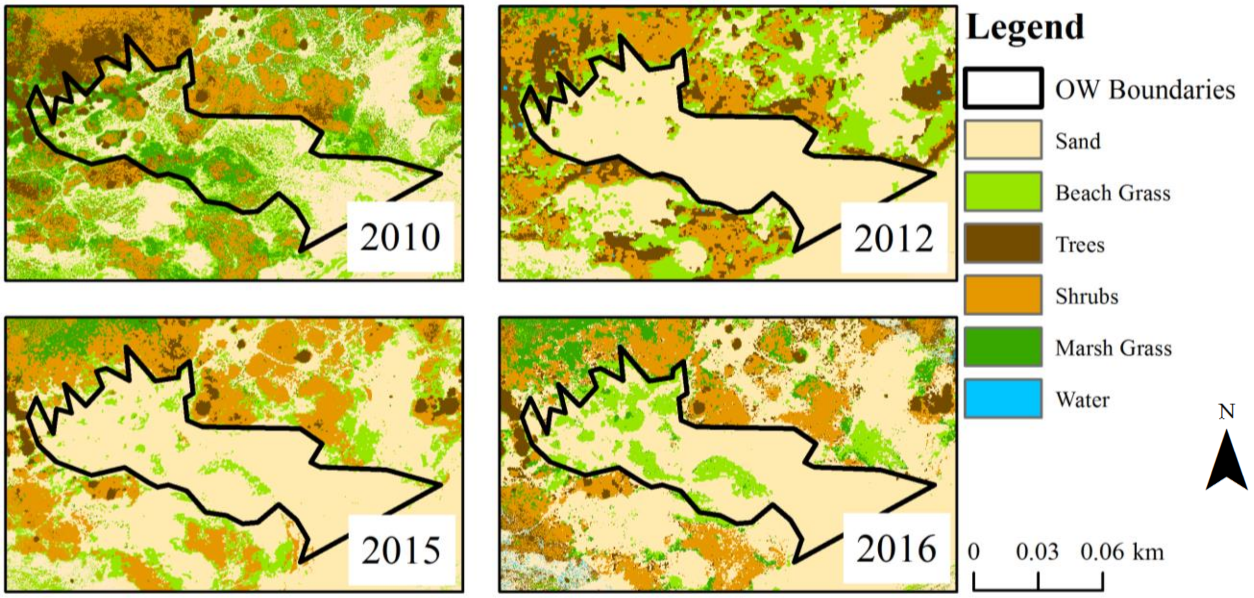

Overwash fan #7

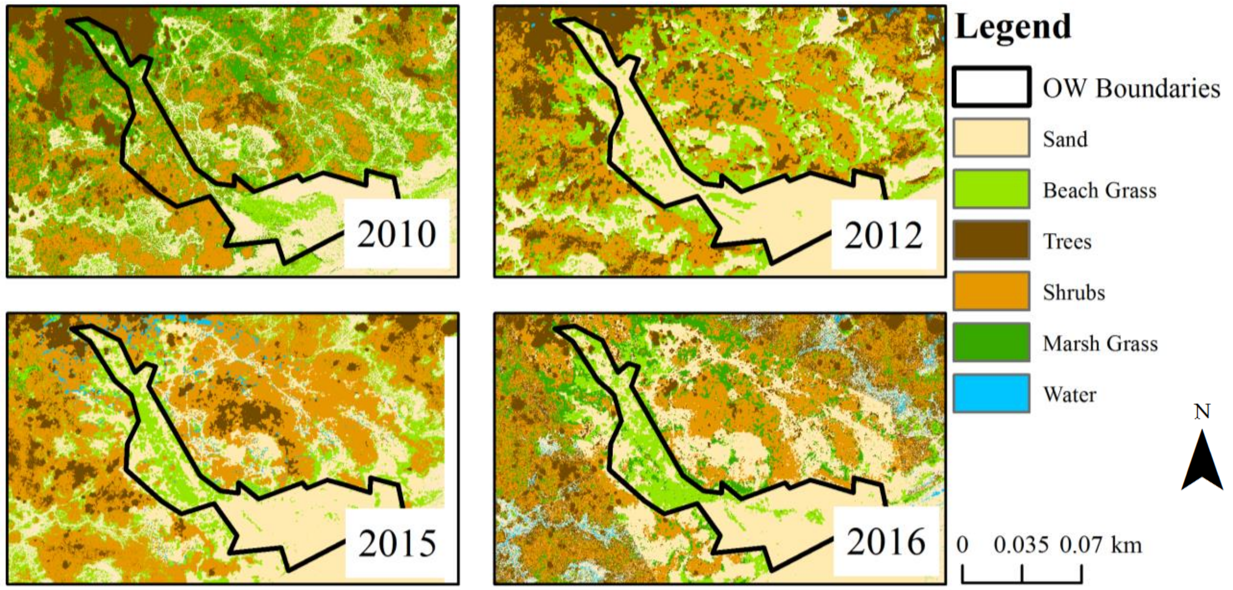

Overwash fan #8

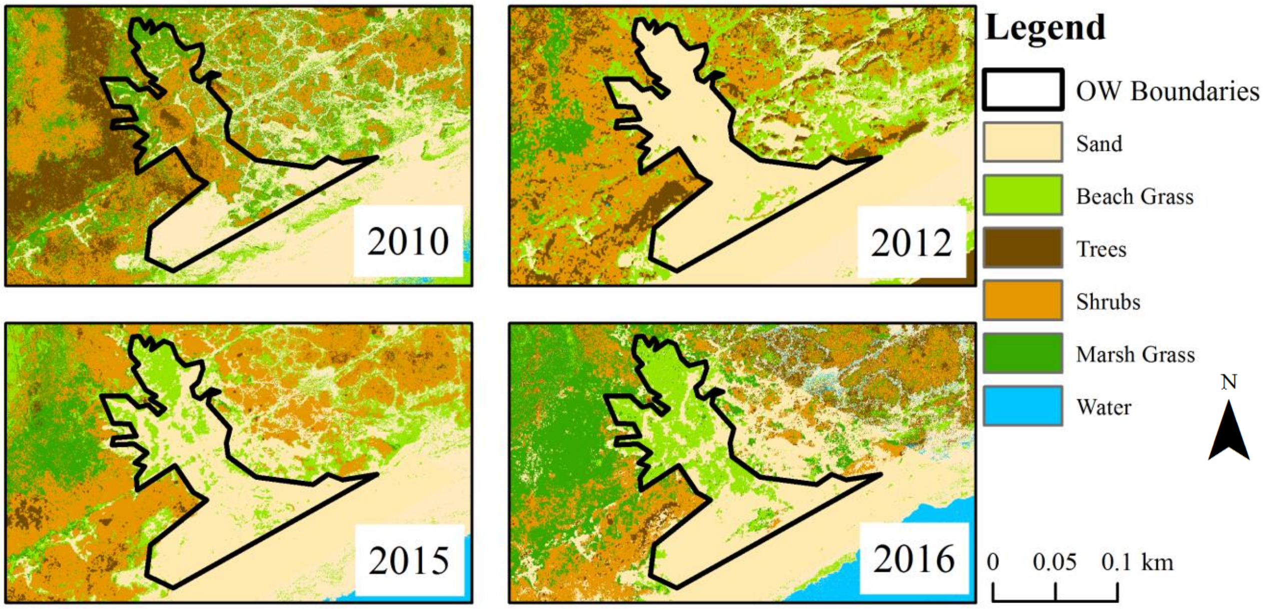

Overwash fan #9

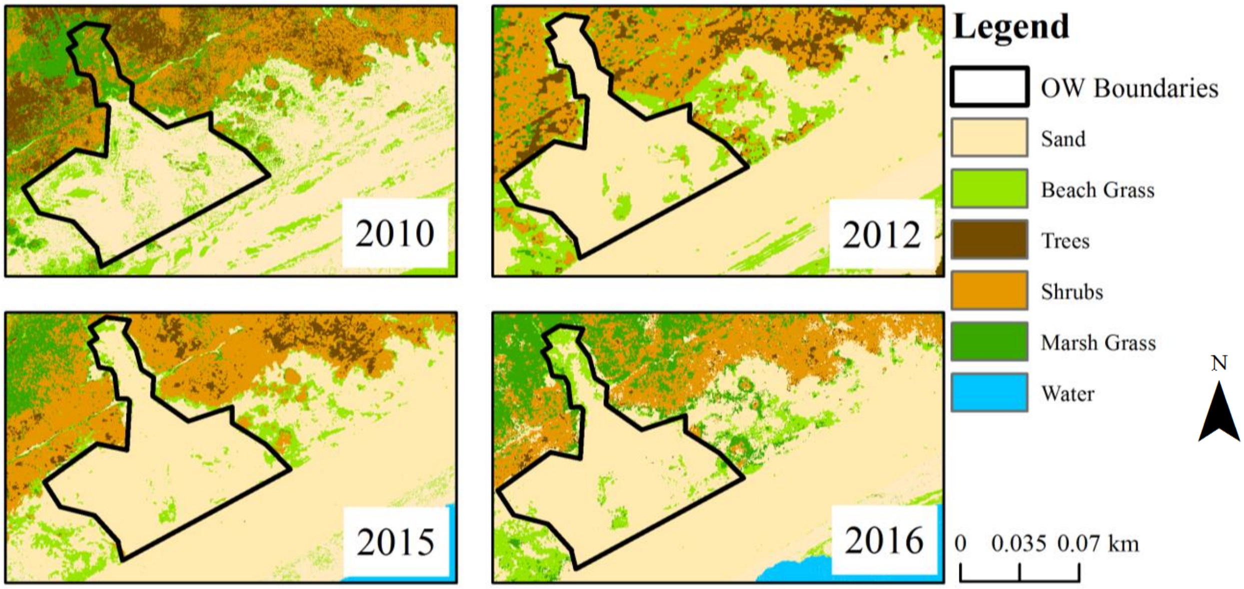

Normalized Difference Vegetation Index calculated for nine areas in the Otis Pike Fire Island High Dune Wilderness Area, New York that were overwashed by Hurricane Sandy from 2012 (before Hurricane Sandy) to 2017 using Landsat 7 ETM+ Surface Reflectance imagery.

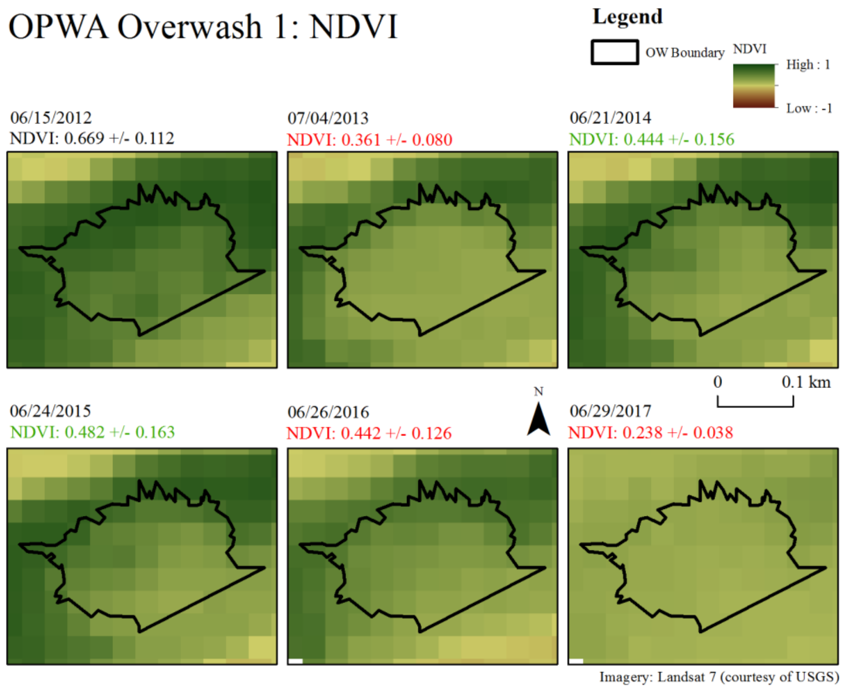

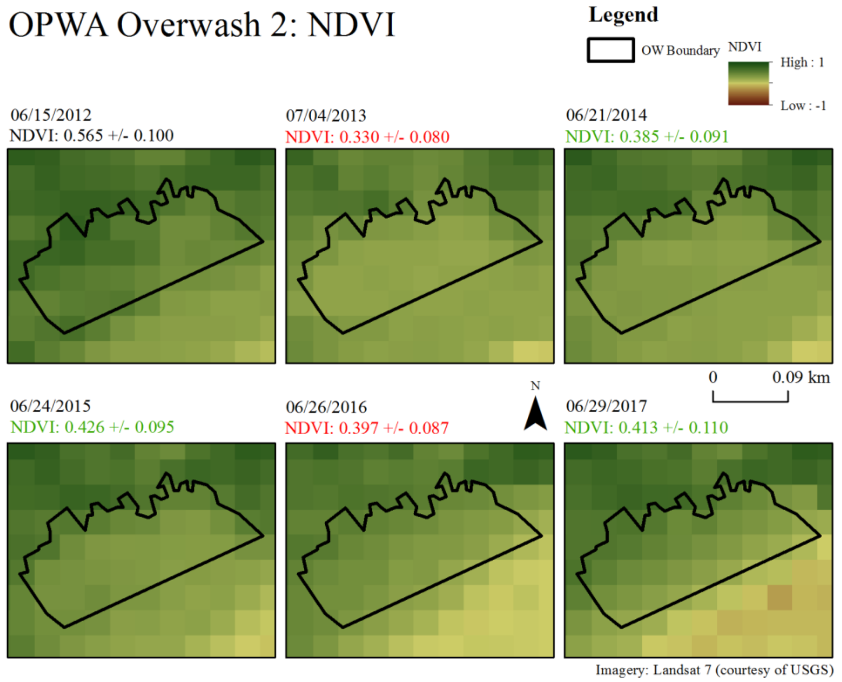

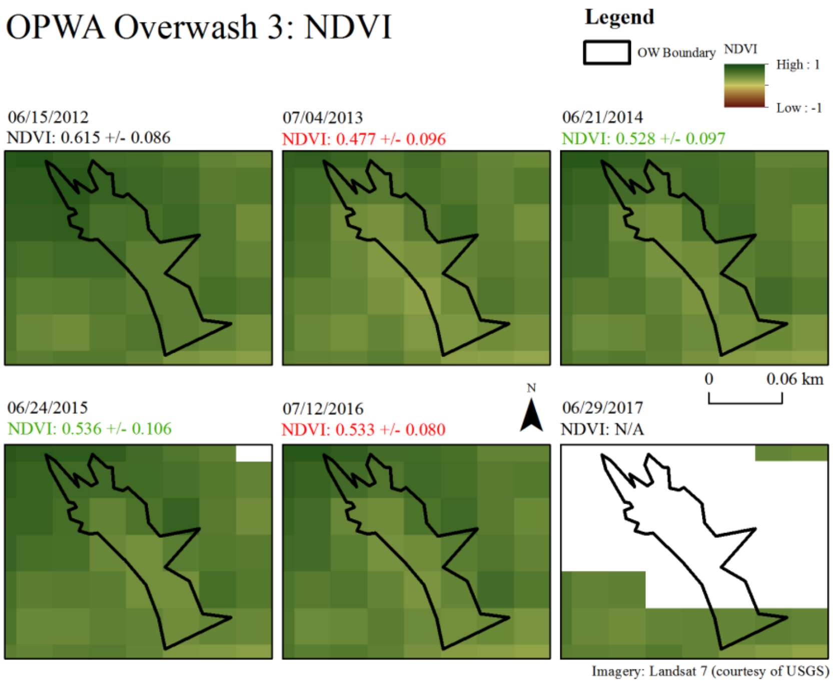
OPWA Overwash 3: NDVI
Legend
OW Boundary
NDVI
High : 1
Low : -1
06/15/2012
NDVI: 0.615 +/- 0.086
07/04/2013
NDVI: 0.477 +/- 0.096
06/21/2014
NDVI: 0.528 +/- 0.097
0
0.06 km
N
06/24/2015
NDVI: 0.536 +/- 0.106
07/12/2016
NDVI: 0.533 +/- 0.080
06/29/2017
NDVI: N/A
Imagery: Landsat 7 (courtesy of USGS)

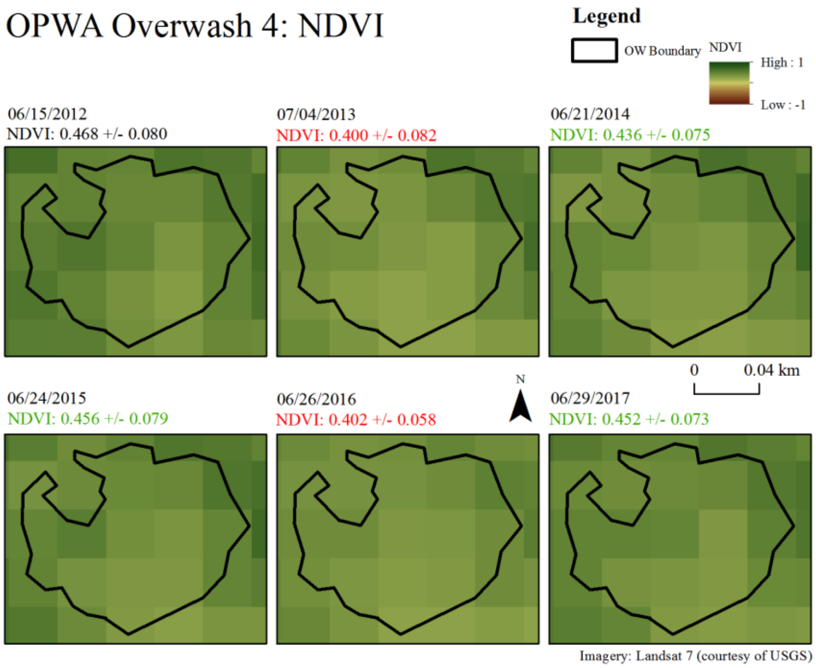
OPWA Overwash 4: NDVI
Legend
OW Boundary
NDVI
High : 1
Low : -1
06/15/2012
NDVI: 0.468 +/- 0.080
07/04/2013
NDVI: 0.400 +/- 0.082
06/21/2014
NDVI: 0.436 +/- 0.075
0
0.04 km
N
06/24/2015
NDVI: 0.456 +/- 0.079
06/26/2016
NDVI: 0.402 +/- 0.058
06/29/2017
NDVI: 0.452 +/- 0.073
Imagery: Landsat 7 (courtesy of USGS)

OPWA Overwash 5: NDVI

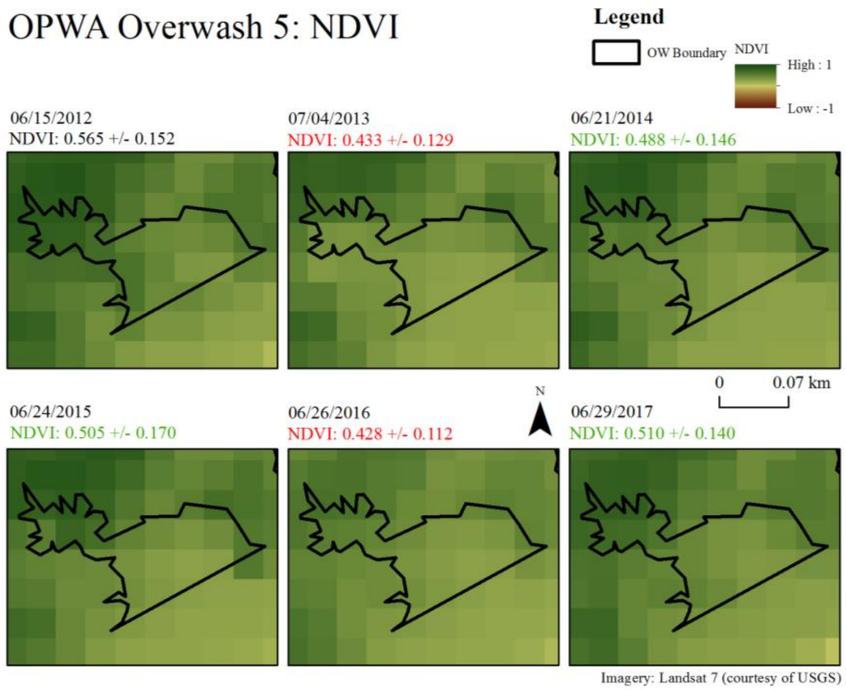

OPWA Overwash 6: NDVI

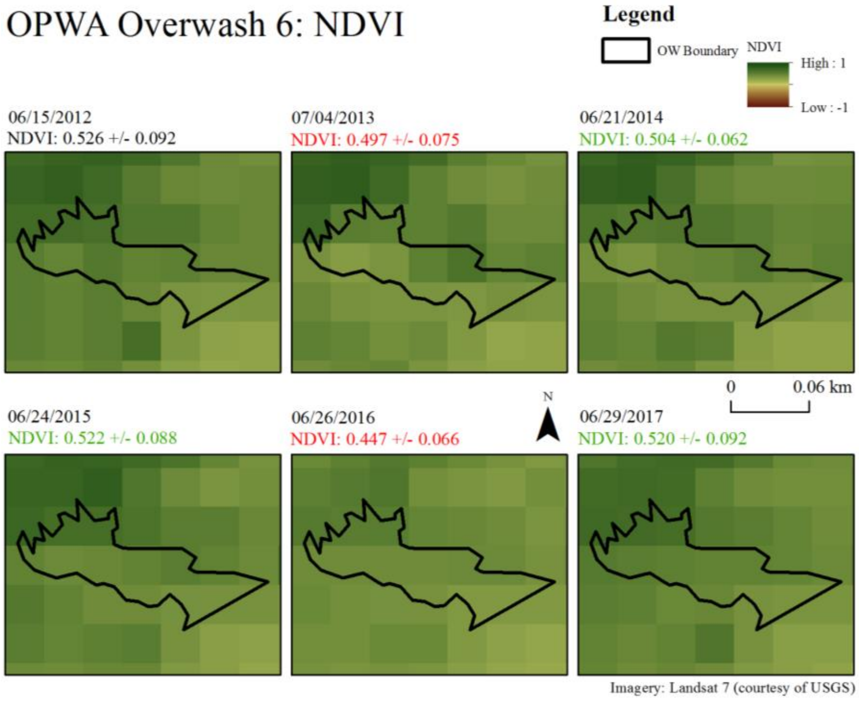

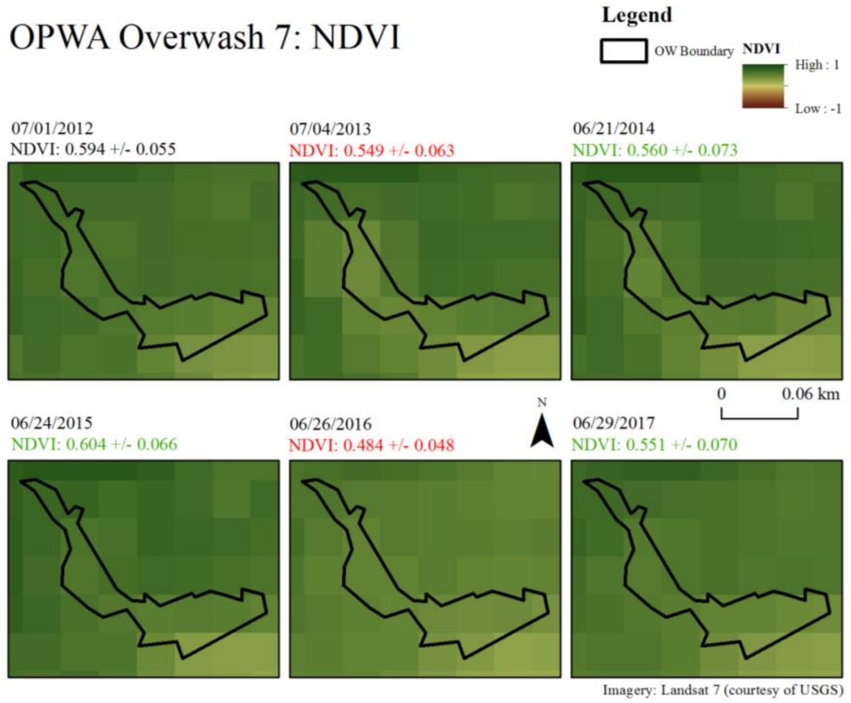
OPWA Overwash 7: NDVI
Legend
OW Boundary
NDVI
High : 1
Low : -1
07/01/2012
NDVI: 0.594 +/- 0.055
07/04/2013
NDVI: 0.549 +/- 0.063
06/21/2014
NDVI: 0.560 +/- 0.073
0
0.06 km
N
06/24/2015
NDVI: 0.604 +/- 0.066
06/26/2016
NDVI: 0.484 +/- 0.048
06/29/2017
NDVI: 0.551 +/- 0.070
Imagery: Landsat 7 (courtesy of USGS)

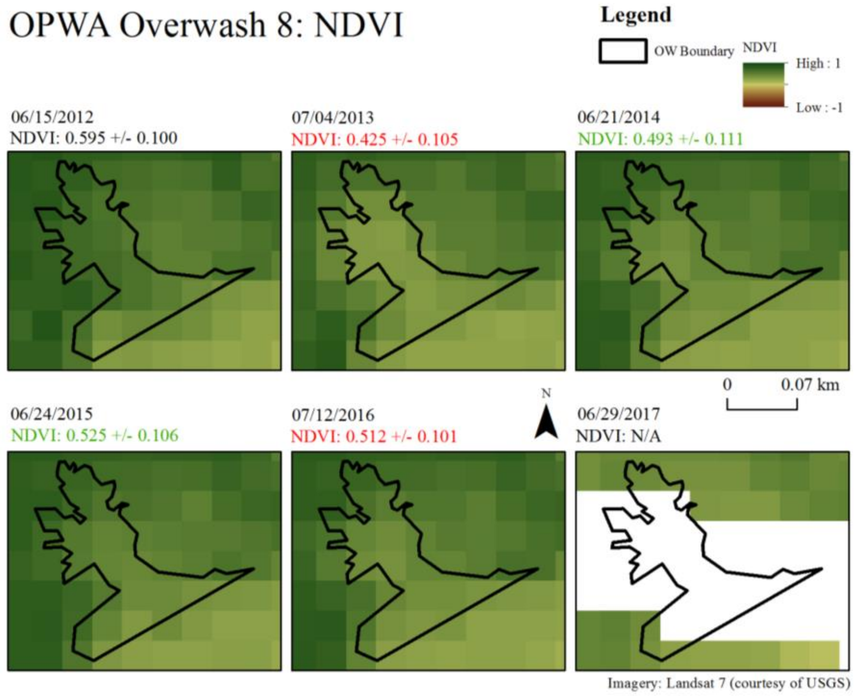
OPWA Overwash 8: NDVI
Legend
OW Boundary
NDVI
High : 1
Low : -1
06/15/2012
NDVI: 0.595 +/- 0.100
07/04/2013
NDVI: 0.425 +/- 0.105
06/21/2014
NDVI: 0.493 +/- 0.111
0
0.07 km
N
06/24/2015
NDVI: 0.525 +/- 0.106
07/12/2016
NDVI: 0.512 +/- 0.101
06/29/2017
NDVI: N/A
Imagery: Landsat 7 (courtesy of USGS)

OPWA Overwash 9: NDVI

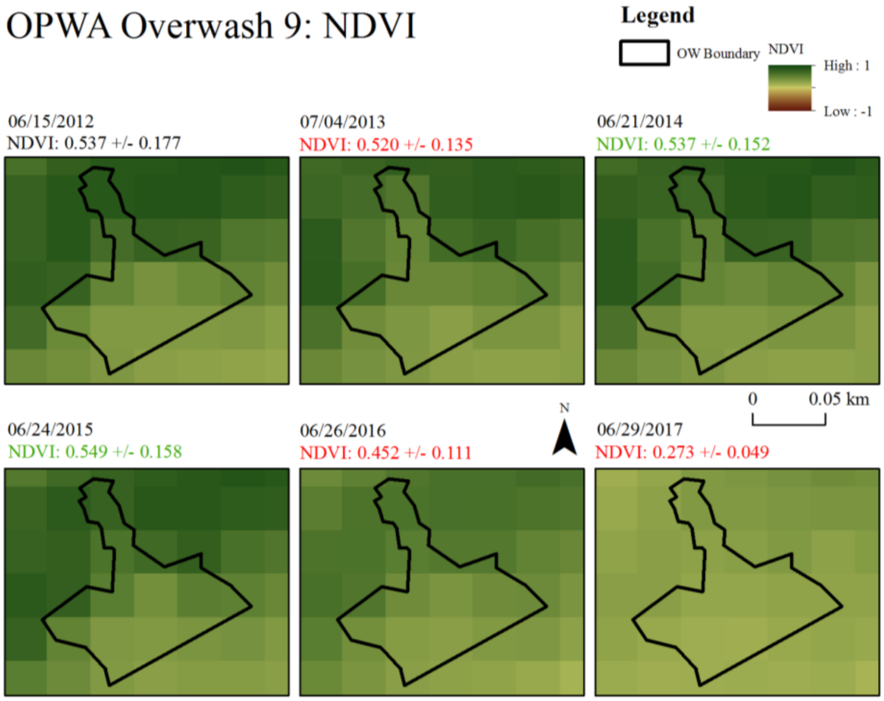

Imagery: Landsat 7 (courtesy of USGS)
